# 

**Published:** 1915-07-03

**Authors:** 


### CONTENTS.

PAGE

**Hospitals and General Practitioners** ... ... 281

**Hospitals and the Spirit Tax** ... ... ... 282

**Hospital and Institutional News** ... ... ... 283

Soldiers and Ward Discipline—Governors' Votes and Governors' Guineas—Gift of Twenty-Five Beds to a Dublin Hospital—Australian Hospitals and the War—The New Research Institute at Melbourne—The Transformation of Livingstone College—The Public and the Ambulance Service—Indian Applicants for Medical Posts—The Annual Festival of the Order of St. John—The Root Difficulty in Insurance Sanatoria—County Councils and Insurance Committees—Board-Room Photographs—Parents and School Medical Inspection—The Modern Girl's Education—Conditional Gifts of Cash in War-Time—Accepting Medical Assistants on Trust—Children and a Public Health Committee—The Hospital Ideal in Australia—Recognition of an Infirmary Medical Superintendent—French Red Cross and the Dardanelles—The Official Respirator—The Wounded up the Thames—The New Hospital at Englefield Green and its Promoters—The Non-Panel Movement—This Week's Drug Market—To Our Readers.

**Voluntary Hospitals and Military Hygiene** ... 289

By H. S. Souttar, F.R.C.S.

**Notes by a Surgeon on Active Service...** ... 290

Applications to Open Wounds—Morphia Injections—Thirst on the March—Enteric Inoculation After-effects.

**Hospital Ships and the Right of Search** ... 291

The Practical Lesson of the *Ophelia* Case.

**Round the World** ... ... ... ... ... 292

Fellow-Workers and the Roll of Honour.

**The Discharge of Tuberculous Soldiers from the French Army** ... ... ... ... 296

PAGE

**National Association for the Feeble-Minded...** 293

The Annual Conference and Exhibition.

**Sir Bryan Donkin's Address** ... ... ... 294

The Test of Conduct and Difficulty of Definition.

**Mental Tests in Childhood** ... ... ... 295

The Causes of Success and Failure.

**The Educative Work of the School Doctor** ... 297

II. Lantern Slides for Parents' Lectures.

**The Editor's Letter-Box** ... ... ... ... 298

Hospitals and Spirit Tax—A Reduction in a Drug Account.

**Presentation for War Service** ... ... ... 298

**Doctors' Wills** ... ... ... ... ... ... 298

**The Institutional Library** ... ... ... 299

The Medical Annual and the War.

Lay Workers and Infant Mortality.

"Kill that Fly."

Water Analysis and Purification.

The Dispenser's Companion.

**Gifts and Bequests** ... ... ... ... ... vi

**Institutional Needs** ... ... ... ... ... vi

**The Book Market** ... ... ... ... ... vi

**The Institutional Worker** ... ... (Suppl.) 1

National Insurance Work: Answers to May Question.

Question for July.

Enquiries and Answers.

The Sick and in Need: Hospital for Gentlewomen in Reduced Circumstances—Home of Rest for Ladies

Employment and Training: Asylums in Yorkshire—Cottage Nursing.

Practical Matters: Refrigeration: the Principles of the Cold Store.

War Matters: Nursing the Wounded—London Military Hospitals.

